# Synthesis of Silicon and Germanium Containing Heteroaromatic Sulfides as Cholesterol Level Lowering and Vasodilating Agents

**DOI:** 10.1155/MBD.2001.85

**Published:** 2001

**Authors:** Kira Rubina, Edgars Abele, Pavel Arsenyan, Ramona Abele, Maris Veveris, Edmunds Lukevics

**Affiliations:** Latvian Institute of Organic Synthesis 21 Aizkraukles street Riga LV-1006 Latvia

## Abstract

Silicon and germanium containing heteroaromatic sulfides have been prepared using phase transfer catalytic (PTC) system thiol / Si or Ge containing alkyl halide / solid KOH / 18- crown-6 / toluene. The target sulfides were isolated in yields up to 92 %. It has been found that 2-{[dimethyl (β-triethylgermylethyl)-silylmethyl]thio}-1-methylimidazole and 2-{[dimethyl(β-triphenylsilylethyl) silyl-methyl]thio}benzothiazole are the most active cholesterol level lowering and vasodilating agents.


### SYNTHESIS OF SILICON AND GERMANIUM CONTAINING HETEROAROMATIC SULFIDES AS CHOLESTEROL LEVEL LOWERING AND VASODILATING AGENTS

Kira Rubina, Edgars Abele, Pavel Arsenyan, Ramona Abele, Maris Veveris, Edmunds Lukevics*

Latvian Institute of Organic Synthesis, 21 Aizkraukles street, Riga, LV-1006, Latvia < kira@osi.lv>

### ABSTRACT

Silicon and germanium containing heteroaromatic sulfides have been prepared using phase transfer catalytic (PTC) system thiol / Si or Ge containing alkyl halide / solid KOH / 18-crown-6 / toluene. The target sulfides were isolated in yields up to 92 %. It has been found that 2-{[dimethyl(β-triethylgermylethyl)-silylmethyl]thio}-1-methylimidazole and 2-{[dimethyl(β-triphenylsilylethyl)silyl-methyl]thio}benzothiazole are the most active cholesterol level lowering and vasodilating agents.

### INTRODUCTION

Coronary heart desease (CHD) remains the leading cause of death in the industrialized countries. The primary cause of CHD is atherosclerosis, a disease characterized by the deposition of lipids in the arterial vessel wall, resulting in a narrowing of the vessel passages and ultimately hardening the vascular system.

Atherosclerosis as manifested in its major clinical complication, ischaemic heart disease, is thought to begin with local injury to the arterial endothelium followed by proliferation of arterial smooth muscle cells from the medial layer to the intimal layer along with deposition of lipid and accumulation of foam cells in the lesion. As the atherosclerotic plaque develops, it progressively occludes more and more blood vessel and can eventually lead to ischaemia or infarction. Therefore, it is desirable to provide a method of inhibiting the progression of atherosclerosis in patients.

Elevated cholesterol levels are also associated with a number of disease states. Therefore it is desirable to provide a method for reducing plasma cholesterol in patients with, or at risk of developing restenosis, angina, cerebral arteriosclerosis, and xanthoma.

Heterocyclic sulfides exhibit a wide spectrum of activity on heart and blood circulatory system. For example, pyridine sulfides display cholesterol acyltransferase inhibitor [1], blood sugar reduction [2], vasodilating [3-7], vasopressing [8], antihypertensive [4, 5, 7, 9, 10], hypotensive [6], hypoglycemic [11-13], anticholesteremic [14], euglicemic and hypolipidemic [15], cardiotonic [16], cardiovascular and cardioprotective [17] activities. Quinoline sulfides exhibit vasodilating [18-24], antihypertensive [18, 19], hypotensive [23, 30], euglicemic and hypolipidemic [15], antithrombotic [31, 32] and cardiotonic [32] activities. Thiazole and benzothiazole sulfides display vasodilating [33-35], and hypotensive [35] activities and may be used as hypolipemics and in treatment of arteriosclerosis [36]. Imidazole sulfides increase high density lipoprotein cholesterol over lipid fractions [37] and were used in the treatment of atherosclerosis [38]. Hypoglycemic activity of N-{4-[2-(pyrazole-1-carbonylamino)ethyl]benzenesulfonyl}urea [39] and vasodilating activity of 6-quinazolinesulfonyl derivatives [40] are evaluated too.

The silicon and germanium containing heteroaromatic sulfides as cholesterol level lowering and vasodilating agents have not been studied. In this view the silyl and germyl S-substituted derivatives of N- and S-heterocyclic thiols are of interest as substances with the possible above described activity.

The preparation of the compounds possessing serum cholesterol level lowering property [41] is the aim of our work.

The known methods for the preparation of hetaryl sulfides are based on the reaction of hetaryl thiols with alkyl or aryl halides in the $K_2CO_3$ / $Me_2CO$ [42], NaOMe / DMF [43] or NaH / $Me_2SO_4$ [44] systems. Recently we described two simple phase transfer catalytic (PTC) methods for the preparation of hetaryl sulfides in the hetaryl thiol / alkyl halide / solid $K_2CO_3$ / 18-crown-6 / toluene [45] or hetaryl S-acetate / alkyl halide / solid KOH / 18-crown-6 / benzene systems [46].

We have used the first [45] of these methods for the preparation of Si and Ge derivatives of N-methylimidazole, pyridine, quinoline, benzothiazole, and purine sulfides to test their serum cholesterol lowering and vasodilating activities.

### MATERIALS AND METHODS

### *CHEMISTRY*

$^1$H NMR spectra were recorded on a Varian 200 Mercury instrument using $CDCl_3$ as solvent and hexamethyldisiloxane (HMDSO) as internal standard. Mass spectra were registered on a GC-MS HP 6890 (70 eV). GC analysis was performed on a Chrom-5 instrument equipped with flame-ionization detector using glass column packed with 5 % OV-101 / Chromosorb W-HP (80-100 mesh) (1.2 m x 3 mm). Bromomethyltrimethylsilane and 3-iodopropyltrimethylsilane were obtained by Grignard reaction [47, 48].

### *HYDROSILYLATION AND HYDROGERMYLATION REACTIONS OF CHLOROMETHYLVINYL DIMETHYLSILANE. GENERAL PROCEDURE.*

A mixture of 0.01 mol of trialkyl(aryl)hydrosilane (germane), 0.01 mol of chloromethylvinyldimethylsilane and $10^{-4}$ M% of Speier's catalyst were stirred for 4 h in a Wheaton vial at room temperature. Processes were controlled by GLC. Products **1a-d** were purified using vacuum distillation or recrystallization from pentane.

### *HALOGEN EXCHANGE REACTION. GENERAL PROCEDURE.*

A mixture of β-(trialkyl(aryl)silyl(germyl)ethyl)silylmethyl chloride **1a-d** (0.01 mol) and 3.5 fold excess of dry sodium iodide was refluxed in acetone for 12 hours. Alkyliodides **2a-d** were purified using vacuum distillation or recrystallization from pentane/methylene chloride.

### *SYNTHESIS OF SILYL AND GERMYL DERIVATIVES OF HETARYL THIOLS. GENERAL PROCEDURE.*

Finely powdered dry $K_2CO_3$ was added to a solution of 10 mmol of thiol (**3 – 9**), 10 mmol of corresponding silyl or germyl substituted alkyl halogenide and 18-crown-6 (1mmol, 264 mg) in 25 ml of toluene. The mixture was refluxed with stirring to achieve the disappearance of the substrates, filtered over the thin silica gel layer and concentrated under reduced pressure. The residue was purified by column chromatography using the mixture benzene-ethyl acetate as eluent.

### *PHARMACOLOGY*

### *CHOLESTEROL LEVEL LOWERING ACTIVITY ASSAY*

Five months old male ICR mice were housed in an air-conditioned room (23° C and 60% humidity) under an artificial 12-hr light-dark cycle (7:00 am – 7:00 pm). Animals were maintained on a basal diet or a high-cholesterol diet supplemented with linoleic acid. The basal diet contained 20% casein, 63.2% sucrose, 10% coconut oil (linoleic acid content: below 1%), 2 % agar, 0.8% vitamin mixture, and 4% salt mixture. The high cholesterol diet consisted of the basal diet plus 1.5% cholesterol and 10% linoleic acid, but the corresponding 2% of sucrose were omitted from the basal diet. We divided 50 mice into 2 groups and gave different diets as follows: for 10 mice – the basal diet (control group), and for 40 – the high cholesterol diet (HC group). Each animal received 5 g of the respective diet daily for 12 weeks. Water was freely available.

9 Weeks after start of the experiment the total and HDH cholesterol were determined in the serum of intact control and high-cholesterol diet animals (3 from each group). High-cholesterol diet group was divided into Cholesterol control group (7 mice) and 5 groups (6 mice each) treated with the studied compounds (10 mg/kg i.p. once a day for 3 weeks).

At the end of the experiment (12 weeks), the mice after overnight fasting were killed by withdrawing blood from the abdominal aorta under ether anaestesia. Serum was separated by centrifugation (1500 g for min), and high-density lipoprotein (HDL) fraction was immediately separated from a portion of the serum by the heparin-manganese precipitation procedure.

Total and HDL cholesterol in the serum and aorta were determined fluroenzymatically.

The results obtained were expressed as the mean ± SEM of data from 5 – 7 mice per group.

### *VASODILATING ACTIVITY ASSAY*

The modified classical method for the experiments on the isolated perfused rabbit ear blood vessels was used. Rabbits of both sexes (2.6-3.3 kg) were killed by *i.v.* injection of pentobarbital sodium (80 mg/kg). The central ear artery was dissected free at the base of the ear and cannulated with polyethylene tubing and perfused at a constant flow (2ml/min) from 4-channel peristaltic pump Gemini (Italy).

The content of the perfusion fluid (mmol) was as follows: NaCl 136.9; KCl 2.68; $CaCl_2$ 1.8; $MgCl_2$ 1.05; $NaHCO_3$ 11.9; $NaH_2PO_4$ 0.42; glucose 5.6 (pH 7.35 at 22°C). Intraluminal inflow perfusion pressure was measured with a Statham P23J transducer and recorded on the physiograph DMP-4B (Narco Bio-Systems, USA). As flow remained constant, the changes in perfusion pressure reflected changes in blood resistance, i.e. the degree of vasoconstriction or relaxation. Vasoconstriction was caused by intraluminal infusion of noradrenaline (10 $\mu$mol). The investigated compounds were dissolved in the perfusion fluid. The relaxant responses to the investigated compounds used in the different concentrations (10 and 50 $\mu$mol) were tested. Responses are expressed as per cent relaxation (% changes in the perfusion pressure) without and with the investigated compounds.

### *ACUTE TOXICITY ASSAY*

The acute toxicity was evaluated in male ICR-JCL mice (19-23 g). The compounds were dissolved-suspended in 0.6% solution of Twin 80 and injected *i.p.* To reduce the number of the used animals and the amount of compounds the maximal dose (400-600 mg/kg, *i.p.*) was used. If possible $LD_{50}$ was calculated when 50% of the animals died.

### RESULTS AND DISCUSSION

### *CHEMISTRY*

Hydrosilylation and hydrogermylation of the chloromethylvinyldimethylsilane with diethylmethylsilane, triethylsilane, triphenylsilane, and triethylgermane have been used to obtain silyl(germyl)containing alkylchlorides **1a – d**. Trialkyl(aryl)silane or germane was added to vinylsilane in the presence Speier's catalyst (0.1 M solution $H_2PtCl_6{\cdot}6H_2O$ in absolute isopropyl alcohol). The reactions were exothermic and gave 1,3-disilyl(germyl)substituted ethanes in good yields (up to 98 %) (Table 1).

It was necessary to transform the silyl(germyl)containing alkylchlorides **1a – d** into iodides due to the low reactivity of alkylchlorides under phase transfer catalysis conditions. The chlorine atom exchange for iodine was performed with NaI in acetone by usual procedure giving the corresponding alkyliodides **2a – d** (in yields up to 74 %) (Table 1).

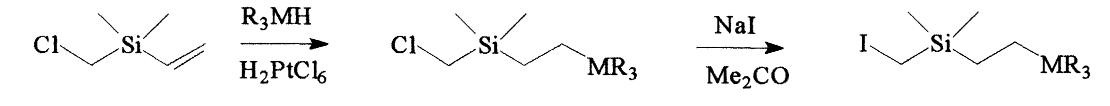

**1a – d** **2a – d**

Table 1. Silyl and germyl containing alkylhalogenides **1a – d**, **2a – d**.

| N° | $R_3M$ | b.p. / (m.p.), °C/mm | Yield, % | MS-GC (m/z) |
|---|---|---|---|---|
| 1a | $Et_2MeSi$ | 106-108/15 | 87 | 236 ($M^+$, 12), 101 (100) |
| 1b | $Et_3Si$ | 114-117/15 | 70 | 250 ($M^+$, 15), 115 (100) |
| 1c | $Ph_3Si$ | (55-56) | 98 | - |
| 1d | $Et_3Ge$ | 122-124/15 | 77 | 296 ($M^+$, 26), 161 (100) |
| 2a | $Et_2MeSi$ | 128-131/15 | 74 | 328 ($M^+$, 5), 101 (100) |
| 2b | $Et_3Si$ | 136-138/15 | 58 | 327 ($M^+$-15, 21), 115 (100) |
| 2c | $Ph_3Si$ | (48-49) | 54 | - |
| 2d | $Et_3Ge$ | 132-134/10 | 66 | 375 ($M^+$-15, 22), 115 (100) |

A simple method for the preparation of silyl and germyl derivatives of the N- and S-heterocyclic thiols was developed. The phase transfer catalytical system solid $K_2CO_3$/18-crown-6/toluene was used. Application of the stronger base (KOH) led to the destruction of alkylating agents. Despite to the mild reaction conditions triphenylsilyl derivative **2c** partly decomposed. Therefore the yield of 2-{[dimethyl(β-triphenylsilylethyl)silylmethyl]thio}benzothiazole **8d** was 13%.

The aimed substances were obtained with good chemical yields (up to 92%) in a short time under mild conditions (Table 2).

$$\text{HetSH} \xrightarrow[\text{toluene / reflux}]{\text{RI / K}_2\text{CO}_3\text{ / 18-crown-6}} \text{HetSR}$$

**3 - 9** **3 (a-e) - 9 (a-e)**

The purity of the synthesized compounds was detected with HPLC (<1.5% of impurities). All substances were mobile oils therefore the elemental analysis was performed only for **8a** and **9a**.

The structures and spectral characteristics of synthesized substances are shown in Tables 3 and 4.

Table 2. Synthesis of element containing derivatives of hetaryl thiols (HetSH : RI : $K_2CO_3$ : 18-crown-6 = 1 : 1 : 4 : 0.1 ) *HetSH : RI = 1 : 2; S- and N-disubstituted derivatives were obtained.

| Thiol | Het | R | Reaction time, h | Product | Isolated yield, % |
|---|---|---|---|---|---|
| 3 | 2-(1-methylimidazolyl) | $(CH_2)_3SiMe_3$ | 8 | 3a | 61 |
| 3 | 2-(1-methylimidazolyl) | $CH_2SiMe_2(CH_2)_2SiEt_2Me$ | 5 | 3b | 84 |
| 3 | 2-(1-methylimidazolyl) | $CH_2SiMe_2(CH_2)_2GeEt_3$ | 6 | 3e | 93 |
| 4 | 2-pyridyl | $(CH_2)_3SiMe_3$ | 8 | 4a | 66 |
| 5 | 4-pyridyl | $(CH_2)_3SiMe_3$ | 7 | 5a | 62 |
| 6 | 2-quinolyl | $(CH_2)_3SiMe_3$ | 7 | 6a | 53 |
| 7 | 8-quinolyl | $(CH_2)_3SiMe_3$ | 7 | 7a | 33 |
| 8 | 2-benzothiazolyl | $(CH_2)_3SiMe_3$ | 10 | 8a | 65 |
| 8 | 2-benzothiazolyl | $CH_2SiMe_2(CH_2)_2SiEt_2Me$ | 6 | 8b | 83 |
| 8 | 2-benzothiazolyl | $CH_2SiMe_2(CH_2)_2SiEt_3$ | 5 | 8c | 62 |
| 8 | 2-benzothiazolyl | $CH_2SiMe_2(CH_2)_2SiPh_3$ | 5 | 8d | 13 |
| 8 | 2-benzothiazolyl | $CH_2SiMe_2(CH_2)_2GeEt_3$ | 6 | 8e | 73 |
| 9 | 1- and 6-puryl* | $(CH_2)_3SiMe_3$ | 9 | 9a | 35 |

Table 3. $^{1}$H and $^{13}$C NMR data of heteroaromatic sulfides **3a – 9a**

| Sulfide | Structure of HetSR | $^{1}$H NMR, δ (ppm, $CDCl_3$ / HMDSO) | $^{13}$C NMR, δ (ppm, $CDCl_3$ / HMDSO) | |
|---|---|---|---|---|
| | | | Het | SR |
| **3a** |  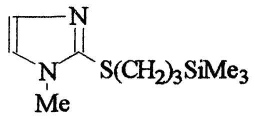 | 0.03 (s, 9H, $Si(CH_3)_3$), 0.91 (m, 2H, $CH_2Si$), 1.64 (m, 2H, $CH_2C\underline{H}_2CH_2Si$), 3.10 (t, 2H, J= 7.0 Hz, $SCH_2$), 3.60 (s, 3H, $NCH_3$), 7.20 (m, 2H, imidazole protons) | 121.86 (C-5), 129.13 (C-4), 142.02 (C-2), 33.11 ($NCH_3$) | -1.81, 16.07, 24.53, 37,81 |
| **3b** |  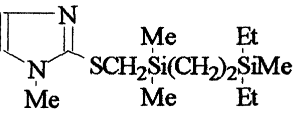 | - 0.10 (s, 3H, $Et_2SiC\underline{H}_3$); 0.10 (s, 6H, $Si(CH_3)_2$); 0.46 (m, 8H, $CH_2Si$); 0.90 (m, 6H, $SiCH_2C\underline{H}_3$); 2.42 (s, 2H, $SCH_2$);, 3.57 (s, 3H, $NCH_3$); 6.88 (d, 1H, J=1.2 Hz, H-5); 7.02 (d, 1H, J= 1.2 Hz, H-4) | 121.79 (C-5), 128.79 (C-4), 144.69 (C-2), 32.83 ($NCH_3$) | -4.13, 4.46, 4.60, 17.07 |
| **3e** |  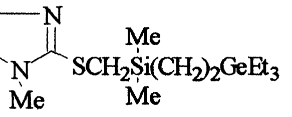 | 0.10 (s, 6H, $Si(CH_3)_2$); 0.72 (m, 10H, $CH_2Si$ and $CH_2Ge$);; 0.99 (t, 9H, J= 7.4 Hz, $GeCH_2C\underline{H}_3$); 2.43 (s, 2H, $SCH_2$); 3.57 (s, 3H, $NCH_3$); 6.87 (d, 1H, J=1.2 Hz, H-5); 7.02 (d, 1H, J= 1.2 Hz, H-4) | 121.76 (C-5), 128.78 (C-4), 144.65 (C-2), 32.79 ($NCH_3$) | -4.16, 3.19, 3.37, 8.61, 8.93, 17.04 |
| **4a** |  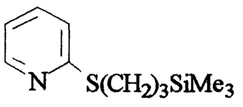 | 0.13 (s, 9H, $Si(CH_3)_3$), 0.72 (m, 2H, $CH_2Si$), 1.80 (m, 2H, $CH_2C\underline{H}_2CH_2Si$), 3.27 (t, 2H, J= 7.6 Hz, $SCH_2$), 7.00 (m, 1H, 5-H), 7.25 (m, 1H, 3-H), 7.91(m, 1H, 4-H), 8.49 (m, 1H, 6-H) | 118.89 (C-5), 121.19 (C-3) 135.59 (C-4), 149.21 (C-6), 161.32 (C-2) | -1.70, 15.40, 22.60, 33.25 |
| **5a** |  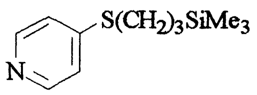 | -0.01 (s, 9H, $Si(CH_3)_3$); 0.66 (m, 2H, $SiCH_2$); 1.70 (m, 2H, $CH_2C\underline{H}_2CH_2$); 2.97 (t, 2H, J=8.0 Hz, $SCH_2$); 7.09 (dd, 2H, $J_1$= 4.0 Hz, $J_2$= 1.4 Hz, H-3,4), 8.38 (dd, 2H, $J_1$= 4.0 Hz, $J_2$= 1.4 Hz, H-2,6) | 120.57 (C-3, C-5), 149.18 (C-2, C-6), 149.46 (C-4) | -1.83, 16.47, 23.40, 34.14 |
| **6a** |  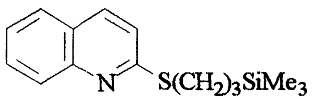 | 0.01 (s, 9H, $Si(CH_3)_3$); 0.76 (m, 2H, $SiCH_2$); 1.80 (m, 2H, $CH_2C\underline{H}_2CH_2$); 3.35 (m, 2H, $SCH_2$); 7.20 – 8.00 (m, 6H, protons of the cycle) | 121.04 (C-3), 125.03 (C-6), 125.87 (C-4a), 127.55 (C-5), 128.01 (C-7), 129.48 (C-8), 135.11 (C-4), 148.36 (C-8a), 159.60 (C-2) | -1.68, 16.59, 24.30, 33.20 |
| **7a** |  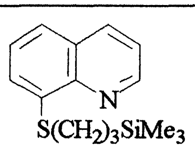 | 0.13 (s, 9H, $Si(CH_3)_3$), 0.93 (m, 2H, $CH_2Si$), 2.47 (m, 2H, $CH_2C\underline{H}_2CH_2Si$), 3.73 (t, 2H, J= 7.0 Hz, $SCH_2$), 7.60, 8.26 and 9.06 (all m, 6H, quinoline cycle protons) | 121.49 (C-3), 123.54 (C-5), 123.76 (C-7), 126.48 (C-6), 128.20 (C-4a), 136.27 (C-4), 138.98 (C-8), 145.51 (C-8a) 149.09 (C-2) | -1.80, 16.66, 23.12, 34.54 |

| | | | | |
|---|---|---|---|---|
| **8a** |  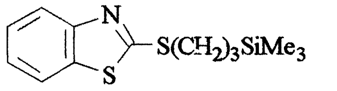 | 0.13 (s, 9H, $Si(CH_3)_3$), 0.81 (m, 2H, $CH_2Si$), 1.84 (m, 2H, $CH_2\underline{CH_2}CH_2Si$), 3.46 (t, 2H, J= 7.4 Hz, $SCH_2$), 7.46 and 7.92 (m, 4H, benzothiazole cycle protons) | 120.87 (C-7), 121.43 (C-4), 124.04 (C-6), 125.95 (C-5), 135.15 (C-7a), 153.38 (C-3a), 167.38 (C-2) | -1.74, 16.37, 24.25, 37.04 |
| **8b** |  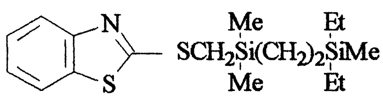 | -0.09 (s, 3H, $Et_2Si\underline{CH_3}$); 0.16 (s, 6H, $Si(CH_3)_2$); 0.51 (m, 8H, $CH_2Si$); 0.90 (t, 6H, J=3.3Hz, $SiCH_2\underline{CH_3}$); 2.61 (s, 2H, $SCH_2$); 7.31 (m, 2H, H-5,6); 7.80 (m, 2H, H-4,7) | 120.87 (C-7), 121.25 (C-4), 123.84 (C-6), 125.93 (C-5), 135.24 (C-7a), 153.58 (C-3a), 170.84 (C-2) | -6.69, -3.93, 4.46, 4.65, 6.99, 7.38, 17.47 |
| **8c** |  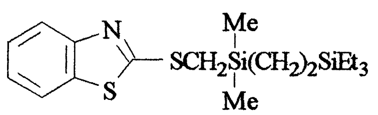 | 0.18 (s, 6H, $Si(CH_3)_2$); 0.52 (m, 10H, $CH_2Si$); 0.91 (m, 9H, $SiCH_2\underline{CH_3}$); 2.63 (s, 2H, $SCH_2$-); 7.31 (m, 2H, H-5,6); 7.79 (m, 2H, H-4,7) | 120.87 (C-7), 121.25 (C-4), 123.84 (C-6), 125.94 (C-5), 135.24 (C-7a), 153.57 (C-3a), 170.86 (C-2) | -3.95, 2.79, 2.85, 3.29, 6.99, 7.44, 17.46 |
| **8d** |  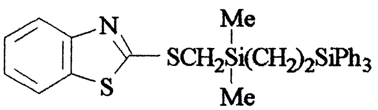 | 0.17 (s, 6H, $Si(CH_3)_2$); 0.76 and 1.34 (both m, 4H, $SiCH_2$); 2.63 (s, 2H, $SCH_2$); 7.35 (m, 2H, H-5,6); 7.50 (m, 15H, Ph); 7.80 (m, 2H, H-4,7) | 120.91 (C-7), 121.29 (C-4), 123.90 (C-6), 125.98 (C-5), 135.26 (C-7a), 153.53 (C-3a), 170.56 (C-2) | -3.86, 5.27, 7.50, 17.32 |
| **8e** |  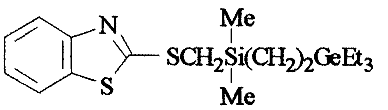 | 0.16 (s, 6H, $Si(CH_3)_2$); 0.68 (m, 10H, $CH_2Si$ and $CH_2Ge$); 0.99 (m, 9H, $GeCH_2\underline{CH_3}$); 2.67 (s, 2H, $SCH_2$); 7.33 (m, 2H, H-5,6); 7.80 (m, 2H, H-4,7) | 120.88 (C-7), 121.26 (C-4), 123.85 (C-6), 125.95 (C-5), 135.24 (C-7a), 153.58 (C-3a), 170.88 (C-2) | -3.91, 3.21,3.43 ,8.72,8.9 9, 17.48 |
| **9a** |  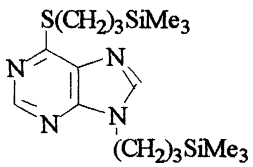 | 0.181 and 1.84 (both s, 9H, $Si(CH_3)_3$), 0.34 (m, 2H, $S(CH_2)_2\underline{CH_2}Si$), 0.57 (m, 2H, $N(CH_2)_2\underline{CH_2}Si$), 1.67 (m, 4H, $CH_2\underline{CH_2}CH_2Si$), 3.26 (m, 2H , $SCH_2$), 4.08 (m, 2H, $NCH_2$), 7.80 (s, 1H, 8-H), 8.57 (s, 1H, 2-H) | 131.41 (C-5),142.48 (C-8), 148.32 (C-4), 151.77 (C-2), 161.40 (C-6) | -1.91, -1.76, 13.63, 16.33, 24.40, 24.83, 32.05, 46.98 |

***PHARMACOLOGY***

*CHOLESTEROL LEVEL LOWERING ACTIVITY*

The compounds **3a**, **b**, **e**; **4a**; **5a**; **6a**; **7a**; **8a**, **b**, **c**, **d**, **e** and **9a** were tested as the cholesterol level-lowering agents.

The Table 5 data show the serum lipid level at the end of the experiment. The high cholesterol in nutrition - Cholesterol group showed the marked increase in the total and LDL cholesterol in comparison to the intact control group. Investigated compounds showed more or less significant protection against increasing in serum total and LDL cholesterol level and the raising of the atherosclerotic coefficient.

It has been found, that 2-{[dimethyl(β-triethylgermylethyl)silylmethyl]thio}-1-methylimidazole (**3e**), 2-{[dimethyl(β-triphenylsilylethyl)silylmethyl]thio}benzothiazole (**8d**), 2-[(γ-trimethylsilylpropyl)thio]quinoline (**6a**) and 2-{[dimethyl(β-methyldiethylsilylethyl)silyl-methyl]thio}benzothiazole (**8b**) exhibit the highest level of activity.

The comparison of compounds containing the different heterocycles showed that 2-(γ-trimethylsilylpropyl)thioquinoline **6a** was more active than thiopyridine analogue **4a**. The 2-[(1-N-methyl)thio]imidazole derivative **3b** was more active than derivative **8b**. Holesterol level lowering effect induced by 2-{[dimethyl(β-triethylgermylethyl)silylmethyl]thio}-1-methylimidazole **3e** was higher than in the case of 2-{[dimethyl(β-triethyl-germylethyl)silylmethyl]thio}benzothiazole **8e**.

The cholesterol level lowering activity depends also on the substituent position in the ring: the 2-position in the pyridine and quinoline is more preferable. Thus 2-[(γ-trimethylsilylpropyl)thio] derivatives of pyridine **4a** and quinoline **6a** are more active than the similar derivatives of 4-pyridine **5a** and 8-quinoline **7a**.

Table 4. Mass spectra of heteroaromatic sulfides

| Sulfide | m/z (relative intensity, %) |
|---|---|
| 3a | 228 ($M^+$, 5), 213 ($M^+$-Me, 10), 181 (17), 171 (39), 114 (100), 73 (49), 72 (15), 59 (11), 45 918), 43 (10), 41 (13) |
| 3b | 313 ($M^+$-1, <1), 187 (8), 186 (14), 185 (100), 73 (13), 59 (9), 45 (13) |
| 3e | 359 ($M^+$- Me, <1), 187 (9), 186 (14), 185 (100) |
| 4a | 225 ($M^+$, 4), 210 ($M^+$-Me, 15), 182 (12), 178 (16), 168 (37), 138 (17), 125 (19), 124 (12), 112 (16), 111 (100), 78 (22), 73 (59), 59 (11), 45 (22), 43 (14) |
| 5a | 225 ($M^+$, 5), 211 (18), 210 ($M^+$-Me, 97), 183 (10), 169 (10), 168 (58), 78 (10), 73 ($SiMe_3$, 100), 59 (15), 51 (16), 45 (23), 43 (13), 39 (12) |
| 6a | 275 ($M^+$, 3), 200 (10), 228 (10), 218 (20), 188 (16), 175 (21), 161 (100), 128 (40), 101 (13), 73 (35), 45 (14) |
| 7a | 275 ($M^+$, 4), 260 ($M^+$-Me, 10), 242 (23), 218 (23), 188 (48), 175 (28), 174 (100), 161 (50), 142 (10), 130 (11), 129 (24), 73 (59), 45 (23), 43 (10) |
| 8a | 281 ($M^+$, 4), 266 ($M^+$-Me, 8), 234 (18), 224 (25), 168 (12), 167 (100), 73 (69), 59 (10), 45 (24), 43 (10) |
| 8b | 352 ($M^+$- Me, 2), 239 (18), 238 (100), 73 (20), 59 (13), 45 (17) |
| 8c | 366($M^+$, 2), 240 ((15), 239 (18), 238 (100), 87 (10), 59 (15) |

Table 5. Effect of hetaryl sulfides on the lipoprotein level and the atherogenicity coefficient (K) on mice with the high cholesterol in nutrition

| Group | Cholesterol, mg/dl | | | K |
|---|---|---|---|---|
| | Total | HDL | LDL | |
| Cholesterol | 120.8 ± 21.3** | 72.2 ± 13.6 | 48.6 ± 15.7** | 0.695 ± 0.266*** |
| 3a | 105.5 ± 13.9* | 72.6 ± 8.5 | 32.9 ± 21.4* | 0.453 ± 0.327** |
| 3b | 95.0 ± 16.0 | 72.2 ± 8.2 | 22.8 ± 12.6* | 0.316 ± 0.168* |
| 3e | 93.3 ± 13.8 | 82.3 ± 14.0 | 10.9 ± 4.6 | 0.137 ± 0.063 |
| 4a | 106.4 ± 22.0* | 77.4 ± 14.2 | 29.0 ± 16.4* | 0.375 ± 0.228* |
| 5a | 103.6 ± 14.6* | 69.9 ± 5.6 | 33.7 ± 14.2* | 0.486 ± 0.203** |
| 6a | 96.6 ± 20.1 | 75.7 ± 18.3 | 20.9 ± 8.6 | 0.291 ± 0.131* |
| 7a | 86.7 ± 15.2 | 65.36 ± 17.2 | 21.3 ± 9.8 | 0.327 ± 0.170* |
| 8a | 84.2 ± 15.8 | 65.6 ± 7.2 | 18.7 ± 13.6 | 0.285 ± 0.210 |
| 8b | 92.3 ± 19.3 | 71.4 ± 8.8 | 20.8 ± 11.0* | 0.283 ± 0.124* |
| 8c | 87.3 ± 11.9 | 65.4 ± 14.3 | 21.8 ± 7.2* | 0.357 ± 0.162* |
| 8d | 82.7 ± 20.8 | 71.4 ± 16.2 | 11.3 ± 10.7 | 0.157 ± 0.137 |
| 8e | 96.8 ± 16.0 | 67.8 ± 5.0 | 29.0 ± 13.2* | 0.424 ± 0.191* |
| 9a | 109.5 ± 26.30* | 70.3 ± 8.5 | 39.3 ± 28.3* | 0.559 ± 0.459** |
| Intact Control | 81.8 ± 5.9 | 77.7 ± 10.4 | 4.1 ± 6.0 | 0.053 ± 0.092 |

* P>0.05 vs Intact Control
** P>0.01 vs Intact Control
*** P>0.001 vs Intact Control

The study of 2-thiobenzothiazole derivatives showed that their activity increased as follows: 2-{[dimethyl(β-triethylgermylethyl)silylmethyl]thio}benzothiazole **8e** << 2-{[dimethyl-(β-triethylsilylethyl)silylmethyl]thio}benzothiazole **8c** < 2-[(3-trimethylsilylpropyl)thio]benzo-thiazole **8a** ≈ 2-{[dimethyl(β-methyldiethylsilylethyl)silylmethyl]thio}benzothiazole **8b** < 2-{[dimethyl(β-triphenylsilylethyl)silylmethyl]thio}benzothiazole **8d**.

*VASODILATING ACTIVITY*

Vasodilating activity of ten substances **3a**, **b**, **e**; **6a**; **8a**, **b**, **c**, **d**, **e** and **9a** has been studied (Table 6).

We have found that imidazole derivatives **3a**, **b**, **e** exhibit high vasodilating activity in experiments *in vivo*. The most active was 2-{[dimethyl(β-triethylgermylethyl)silylmethyl]thio}-1-

methylimidazole (**3e**). The exchange of imidazole ring to benzothiazole, quinoline or purine decreases the vasodilating activity. Unexpectedly, the replacement of triethylsilyl group in the compound **8c** by triphenyl group (**8d**) changes the action type from strong vasodilation to medium vasoconstriction.

Table 6. Vasodilating activity of hetaryl sulfides on rabbit's ear artery

| Compound | Concentration, μM | Relaxation ("-"contraction), % |
|---|---|---|
| **3a** | 10 | 13* |
| | 50 | 36* |
| **3b** | 10 | 12* |
| | 50 | 26* |
| **3e** | 10 | **22*** |
| | 50 | **38*** |
| **6a** | 10 | 6 |
| | 50 | 17 |
| **8a** | 10 | **3** |
| | 50 | **-19** |
| **8b** | 10 | 10 |
| | 50 | 20 |
| **8c** | 10 | **18*** |
| | 50 | **28*** |
| **8d** | 10 | **-12** |
| | 50 | **-20** |
| **8e** | 10 | 3 |
| | 50 | 10 |
| **9a** | 10 | 11 |
| | 50 | 22 |
| Control/Solvent | - | 1 |

* P < 0.05 vs Control

Compounds 2-[(3-trimethylsilylpropyl)thio]benzothiazole (**8a**) and 2-{[dimethyl(β-triphenylsilylethyl)silylmethyl]thio}benzothiazole (**8d**) have shown the highest contraction effect.

### *ACUTE TOXICITY*

The acute toxicity of the substances **3a, b, e**; **6a** and **8a, b, c, d, e,** that were investigated as the cholesterol level lowering and vasodilator agents, was also determined (Table 7).

Table 7. Acute toxicity of the heteroaromatic sulfides

| Compound | LD50 (mg/kg, i.p.) |
|---|---|
| **3a** | 210 (150,0-294,0) |
| **3b** | 200 (152,9-282,98) |
| **3e** | 435 (310,7-609,0) |
| **6a** | > 600 |
| **8a** | 240 (145,5-396,0) |
| **8b** | > 600 |
| **8c** | > 600 |
| **8d** | > 600 |
| **8e** | > 600 |

The studied compounds have a medium toxicity. The most toxic was silyl substituted imidazole sulfide **3b** (200 mg/kg). The replacement of silicon atom by germanium one (**3e**) leads to the decrease of acute toxicity (435 mg/kg). In general, silyl and germyl substituted benzothiazoles (**8b – e**) are less toxic than the corresponding imidazole analogues (**3b, 1h**).

### CONCLUSIONS

The PTC method of the synthesis of heterocyclic silyl- and germylalkylsulfides was elaborated. Thirteen compounds were synthesized and isolated in the yield up to 92%.

They were studied as serum cholesterol level lowering agents. It has been found that silicon and germanium containing heteroaromatic sulfides possess the cholesterol level lowering activity. The 1-methylimidazole, benzothiazole and 2-quinoline derivatives **3e**, **6a**, **8a**, **b**, **e** exhibit the highest level of activity. The substances containing dimethyl(β-triethylgermylethyl)silylmethyl-(**3e**) and dimethyl(β-triphenylsilylethyl)silylmethyl-(**8d**) substituents were the most active.

Ten of the elaborated compounds were tested on the vasodilating activity. It was shown that 1-methylimidazole derivatives **3a**, **3e** possess the vasodilating activity.

The compounds containing benzothiazole have the different influence. Compound with dimethyl(β-triethylsilylethyl)silylmethyl-substituent (**8c**) has the relaxation effect. On contrary the 3-trimethylsilylpropyl-(**8a**) and dimethyl(β-triphenylsilylethyl)silylmethyl – (**8d**) benzothiazole derivatives possess the contraction effect.

The toxicity of the studied substances was low. It was shown that germanium derivative **3e** was less toxic than its silicon analogue **3b**. The introduction of the second silicon or germanium atom also decreases acute toxicity of the compound (**8a** and **8b**, **c**, **d**, **e**).

**ACKNOWLEDGEMENT**

The authors thank Mrs. Ilze Sleik_a for the synthesis of trimethylsilylmethyl and γ-trimethylsilylpropyl iodides and Dr. Juris Popelis for the NMR spectra registration.

**REFERENCES:**

1. S.S. Ko, R.G. Wilde, I. Delucca, H. Li, H.S. Kezar III, G.A. Boswell, A.S. Srivastava, U.S. Patent 5,583,147 (1996); *Chem. Abstr.*, **126**, 117868a (1997).
2. B. Blank, J.G. Gleason, U.S. Patent 3,873,552 (1975); *Chem. Abstr.*, **83**, 9812n (1975).
3. Z.J. Vejdelek, V. Tr_ka, J. Hejny, V. Vy_atova, *Chem. Listy*, **48**, 435 (1954); *Chem. Abstr.*, **49**, 3958f (1955).
4. E. Wehinger, F. Bossert, G. Franckowiak, A. Heise, S. Kazda, H. Meyer, K. Stoepel, R. Towart, Ger. Patent 2,747,513 (1979); *Chem. Abstr.*, **91**, 74480g (1979).
5. C. Materne, Ger. Patent 3,022,030 (1981); *Chem. Abstr.*, **96**, 85553n (1982).
6. S.S. Pharmaceutical Co., Ltd. Jap. Patent 81,123,992 (1981); *Chem. Abstr.*, **96**, 35299p (1982).
7. V.V. Kastron, R. Vitolina, G. Duburs, M. Selga, G. Zarins, N.V. Kondratenko, V.I. Popov, A.A. Kolomeitsev, L.M. Yagupol'skii, France Patent 2,509,727 (1983); *Chem. Abstr.*, **99**, 139778x (1983).
8. K. Kaji, Jap. Patent 6,811,906 (1968); *Chem. Abstr.*, **69**, 106731d (1968).
9. Otsuka Pharmaceutical Factory, Inc. Jap. Patent 58,116,489 (1983); *Chem. Abstr.*, **99**, 158411p (1983).
10. J.J. Baldwin, G.S. Ponticello, Eur. Patent 14,893 (1980); *Chem. Abstr.*, **94**, 103176g (1981).
11. C.E. Berkoff, N.W. DiTullio, J.A. Weisbach, Ger. Patent 2,216,576 (1972); *Chem. Abstr.*, **78**, 16044p (1973).
12. M. Takatani,Y. Sugiyama, T. Kawamoto, K. Adachi, PCT Int. Appl. WO Patent 9,920,632 (1999); *Chem. Abstr.*, **130**, 311794w (1999).
13. Y. Ohara, M. Suzuki, N. Miyachi, K. Kato, K. Ohdoi, T. Kobayashi, K. Shikada, T. Naito, T. Yotsumoto, U.S. Patent 5,955,481 (1999); *Chem. Abstr.*, **131**, 214281q (1999).
14. C.S. Tauro, Eur. Patent 53,681 (1982); *Chem. Abstr.*, **97**, 182217j (1982).
15. B.B. Lohray, V. Bhushan, A.S. Reddy, P.B. Rao, N.J. Reddy, P. Harikishore, N. Haritha, R.K. Vikramadityan, R. Chakrabarti, R. Rajagopalan, K. Katneni, *J. Med. Chem.*, **42**, 2569 (1999).
16. J. Horiuchi, K. Suzuki, M. Ito, Y. Shidori, T. Kato, Jap. Patent 63,201,168 (1988); *Chem. Abstr.*, **110**, 95013h (1989).
17. H.J. Lang, H.W. Kleemann, W. Scholz, U. Albus, Eur. Patent 602,523 (1994), *Chem. Abstr.*, **121**, 157533r (1994).
18. K. Ikezono, S. Fujita, M. Umezato, E. Hosoki, Y. Toba, A. Kusunoki, S. Shitani, *Arzneim.-Forsch.*, **42**, 1200 (1992).
19. A. Birch, R. Davies, L. Maclean, K. Robinson, *J. Chem. Soc., Perkin Trans. 1,* 387 (1994).
20. E. Koltai, G. Zolyomi, P. Komaromy, D. Banfi, T. Szuts, K. Takacs, *J. Labelled Compd. Radiopharm.* **18**, 1107 (1981).
21. T. Kishimoto, H. Kouchi, Y. Kaneda, Jap. Patent 7,420,189 (1974); *Chem. Abstr.*, **81**, 105317j (1974).
22. T. Kishimoto, Jap. Patent 8,000,378 (1980); *Chem. Abstr.*, **93**, 46443v (1980).
23. Tanabe Seiyaku Co., Ltd., Jap. Patent 8,130,991 (1981); *Chem. Abstr.*, **95**, 150717q (1981).
24. Fujisawa Pharmaceutical Co., Ltd., Jap. Patent 5,749,553 (1982); *Chem. Abstr.*, **98**, 215604j (1983).
25. R. Geiger, V. Teetz, B. Schoelkens, Ger. Patent 2,946,909 (1981); *Chem. Abstr.*, **95**, 150470d (1981).

26. D.E. Portlock, Ger. Patent 3,018,545 (1980); *Chem. Abstr.*, **94**, 208725p (1981).
27. L. Jirkovsky, R. Noureldin, *J. Heterocycl. Chem.*, **17**, 449 (1980).
28. M.E. Freed, J.L. Diebold, U.S. Patent 4,251,444 (1981); *Chem. Abstr.*, **95**, 25154c (1981).
29. R.V. Davies, Ger. Patent 3,138,121 (1982); *Chem. Abstr.*, **97**, 23645f (1982).
30. Tanabe Seiyaku Co., Ltd., Jap. Patent 57,134,469 (1982); *Chem. Abstr.*, **98**, 53714r (1983).
31. E. Mueller, Ger. Patent 2,928,583 (1981); *Chem. Abstr.*, **94**, 156773f (1981).
32. E. Mueller, J. Nickl, J. Rosch, B. Narr, W. Haarmann, J.M. Weisenberger, U.S. Patent 4,329,347 (1982); *Chem. Abstr.*, **97**, 144791j (1982).
33. J. Cherqui, Brit. Patent 1,565,885 (1980); *Chem. Abstr.*, **94**, 15713z (1980).
34. J.G. Maillard, P.P.A. Delaunay, J.M.G. Legeai, Eur. Patent 21,940 (1981); *Chem. Abstr.*, **94**, 193317u (1981).
35. S.S.Pharmaceutical Co., Ltd., Jap. Patent 8,209,787 (1982); *Chem. Abstr.*, **96,** 217861y (1982).
36. T. Doll, E. Schacht, E. Hans, E. Schulze, Ger. Patent 2,950,095 (1981); *Chem. Abstr.*, **95,** 132865g (1981).
37. H. Elokdah, T. Sulkowski, D. Cochran, M.-L. McKean, E. Quinet, *Bioorg. Med. Chem. Lett.*, **10**, 1791 (2000).
38. J.T. Billheimer, P.J. Gillies, R.R. Wexler, C.A. Higley, T.P. Maduskuie, Jr., Eur. Pat. 372445 (1990); *Chem. Abstr.*, **114**, 6503k (1991).
39. Z. Brzozowski, S. Magielka, *Acta Pol. Pharm.*, **38**, 521 (1981); *Chem. Abstr.*, **96**, 92190r (1982).
40. H. Hidaka, T. Sone, Y. Sasaki, T. Sugihara, S. Takagi, K. Sako, Eur. Pat. 46,572 (1982); *Chem. Abstr.*, **96**, 217873d (1982).
41. S.J.T. Mao, M.T. Yates, R.A. Parker, US Pat. 5,677,291 (1997); *Chem. Abstr.*, **127**, 3461892z (1997).
42. J. Ehrenfreund, Eur. Pat. 22748 (1981); *Chem. Abstr.*, **95**, 7078b (1981).
43. V. Klimesova, M. Svoboda, K. Waisser, J. Kaustova, V. Buchta, K. Kralova, *Eur. J. Med. Chem.*, **34**, 433 (1999).
44. G. Scheffler, J. Engel, V. Jakovlev, B. Nickel, K. Thiemer, Eur. Pat. 149088 (1985); *Chem. Abstr.*, **103**, 215189z (1985).
45. E. Abele, K. Rubina, R. Abele, I. Sleiksa, E. Lukevics, *Chem. Geterotsikl. Soedin.*, 1197 (1999).
46. E. Abele, R. Abele, J. Popelis, E. Lukevics, *Latv. J. Chem.*, N 2, 61 (1998).
47. J.W. Wilt, C.F. Dockus, *J. Amer. Chem. Soc.*, **92**, 5813 (1970).
48. C.M.Harbordt, D.H.O`Brien, *J.Organomet. Chem.*, **111**(2), 153 (1976).

